# Supplementary material for: Identification of photoperiod-induced specific miRNAs in the adrenal glands of Sunite sheep (Ovis aries)
Source: Front Vet Sci. 2022 Jul 22;9:888207. doi: 10.3389/fvets.2022.888207 (PMC9354845; doi:10.3389/fvets.2022.888207)
Supplement: Supplementary file 1 [file Data_Sheet_1.ZIP › Supplementary file/miRDeep2 file.zip!/miRDeep2 file/Novel_Structure/SPLP42A3/Novel_412.pdf]

Provisional ID	:	NC_019466.2_18692
Score total	:	1.6
Score for star read(s)	:	-1.3
Score for read counts	:	0
Score for mfe	:	1.3
Score for randfold	:	1.6
Score for cons. seed	:	
Total read count	:	127
Mature read count	:	124
Loop read count	:	0
Star read count	:	3

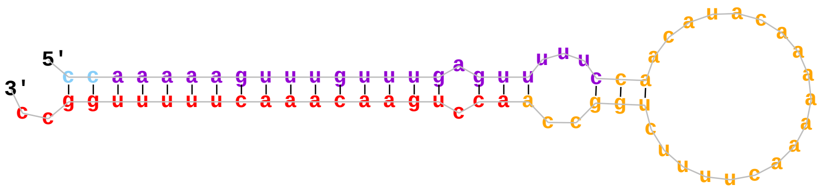

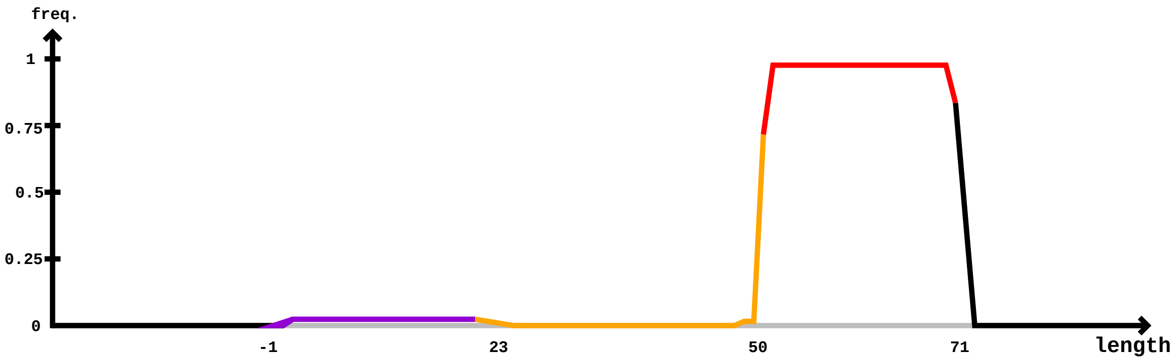


```
                          Star                                       Mature
5'- ggggugcauguauugaauuggccaaaaaguuuguuugaguuuuuccaacauacaaaaaaacuuuucuggccaaccugaacaaacuuuuuggccaaccccaauuuuuugucca   -3'   obs
    ggggugcauguauugaauuggccaaaaaguuuguuugaguuuuuccaacauacaaaaaaacuuuucuggccaaccugaacaaacuuuuuggccaaccccaauuuuuugucca         exp
    (((((.............(((((((((((((((((((.(((...(((...................)))..))).))))))))))))))))))))))))).............   reads   mm   sample
    .......................aaaaaguuuguuugGguuuuu....................................................................   1       1    seq
    .......................aaaaaguuuguuugGguuuuuc...................................................................   2       1    seq
    ......................................................................caaccugaacaaacuuuuuggc....................   2       0    seq
    ........................................................................aUcugaacaaacuuuuuggc....................   16      1    seq
    ........................................................................aUcugaacaaacuuuuuggcc...................   73      1    seq
    .........................................................................Ucugaacaaacuuuuuggcc...................   30      1    seq
    .........................................................................Ucugaacaaacuuuuuggcca..................   3       1    seq
```
